# The failure of community-based health insurance schemes in Tanzania: opening the black box of the implementation process

**DOI:** 10.1186/s12913-021-06643-6

**Published:** 2021-07-03

**Authors:** Ramadhani Kigume, Stephen Maluka

**Affiliations:** 1 Department of History, Political Science & Development Studies, Dar es Salaam University College of Education, P.O.BOX 2329, Dar es Salaam, Tanzania; 2 Institute of Development Studies, University of Dar es Salaam, P.O.BOX 35169, Dar es Salaam, Tanzania

**Keywords:** Community-Based Health Insurance, Health Policy, Health System, Tanzania

## Abstract

**Background:**

Globally, there is increased advocacy for community-based health insurance (CBHI) schemes. Like other low and middle-income countries (LMICs), Tanzania officially established the Community Health Fund (CHF) in 2001 for rural areas; and *Tiba Kwa Kadi* (TIKA) for urban population since 2009. This study investigated the implementation of TIKA scheme in urban districts of Tanzania.

**Methods:**

A descriptive qualitative case study was conducted in four urban districts in Tanzania in 2019. Data were collected using semi-structured interviews, focus group discussions and review of documents. A thematic approach was used to analyse the data.

**Results:**

While TIKA scheme was important in increasing access to health services for the poor and other disadvantaged groups, it faced many challenges which hindered its performance. The challenges included frequent stock-out of drugs and medical supplies, which frustrated TIKA members and hence contributed to non-renewal of membership. In addition, the scheme was affected by poor collections and management of the revenue collected from TIKA members, limited benefit packages and low awareness of the community.

**Conclusions:**

Similar to rural-based Community Health Fund, the TIKA scheme faced structural and operational challenges which subsequently resulted into low uptake of the schemes. In order to achieve universal health coverage, the government should consider integrating or merging Community-Based Health Insurance schemes into a single national pool with decentralised arms to win national support while also maintaining local accountability.

## Introduction

Over the past few decades, the global community has been advocating for Community-Based Health Insurance (CBHI) schemes in order to increase access to healthcare services and thus achieve Universal Health Coverage (UHC). The CBHI is voluntary, non-profit health insurance, normally organized at the community level, mainly targeting those working in the informal sector [1–4]. CBHIs are praised for their ability to reach low-income people and marginalized groups in the society in both rural and urban areas [2, 5, 6]. CBHI follows the principles of insurance including resource pooling, prepayment and risk-sharing and negotiation with other partners in the health system to improve access and quality of healthcare services. It also subscribes to financial protection and responsiveness of health services [3]. Pooling risks across members enrolled in the CBHI scheme reduces the financial burden among the members, as they do not have to incur financial costs to receive healthcare services [5]. Consequently, most recently, many Low and Middle Income countries (LMICs) have included CBHI in their strategies towards UHC which advocates for countries to guarantee all people access to healthcare services without facing financial deprivation [2].

Like other LMICs, Tanzania has been implementing Health Sector Reforms (HSRs) since 1990 s. As part of the HSRs, CBHI schemes were introduced in order to organise and mobilise community initiatives to finance health services. The Community Health Fund (CHF) and Tiba Kwa Kadi (TIKA) are types of CBHI schemes which are currently implemented in Tanzania. CHF was piloted in few districts in Tanzania in 1996 as an initiative to make healthcare services available and affordable to the people residing in rural areas; and those in the informal sector. In 2001, the government enacted the CHF Act and declared CHF a voluntary prepayment health financing mechanism to be rolled out countrywide [7]. TIKA was established in 2009 for the same purpose as CHF but focused only on urban areas [3]. While CHF membership is for the entire household, TIKA is for individual members of the household. The CHF and TIKA receive subsidies from the central government in the form of matching grants.

Following the weakness in the performance of CHF in terms of enrolment, fund management and benefit coverage, the then Ministry of Health and Social Welfare (MoHSW) decided to transfer the management of CHF operations to the National Health Insurance Fund (NHIF) in 2009, as the latter has more experience and expertise in the health industry [8]. The change aimed to harmonise NHIF and CHF management operations, by incorporating CHF management structures within the NHIF in order to improve efficiency and supervision and to increase awareness of the CHF and coverage in line with universal coverage objectives [8]. The NHIF conducted a status assessment to determine national CHF coverage and developed a three-year Action Plan (2009–2012) that guided the operation of the newly established linkage between the NHIF and CHF/TIKA. The specific interventions mentioned in this plan included reviewing the CHF benefit package, registration, and collection mechanisms; introducing claims management and information systems; enhancing the capacity of CHF operations; reviewing user fees to encourage CHF membership; and establishing a Risk Equalization and Reinsurance Fund [8].

From the onset to the recent reviews of evidence, low membership has been persistently observed in CBHI schemes in LMICs [9–11], and this has also been the case in Tanzania [3, 12–15]. Many causes of low membership have been identified; and they include affordability, distance to health facilities, quality of the offered healthcare services [12–15] and trust in scheme management and in healthcare providers [12]. There are also issues with organization and management of the schemes such as provision of benefit packages, response to expectations and information of the target population [11, 13, 14, 16–18]. A recent study in Tanzania indicated that exemption policies and healthcare-seeking behaviour negatively influenced the maximum potential enrolment rate of the CHF scheme. In addition, the study showed that higher revenues from user fees, user fee policies and fund pooling mechanisms motivated healthcare workers to prioritize user fees over CHF revenues [19]. Generally, the assessment of CBHI indicates that apart from a few successful experiences, such schemes suffer from persistent low membership which could be attributed to lower socioeconomic status, poor quality of health care, lack of benefit from the scheme, lack of trust in scheme management and dissatisfaction with scheme services [20].

Many studies conducted in Tanzania have mainly examined rural-based community health insurance and, therefore, there is scanty evidence on how the TIKA scheme is implemented in urban Tanzania. In order to inform policy and practice, there is a need to investigate the implementation of CBHI schemes in urban settings. To that end, this study investigated the implementation of TIKA scheme in urban districts of Tanzania. Understanding facilitators and barriers to the implementation of the urban-based CBHI scheme is important in order to inform the ongoing efforts to achieve Universal Health Coverage.

## Methodology

### Study design and settings

This study adopted a descriptive qualitative case study design [21]. Descriptive case study was considered important because the study aimed to provide a concise but thorough account of the implementation of TIKA scheme in its natural settings. The focus of this study was mainly to describe the implementation of TIKA scheme in urban settings in Tanzania. The study was conducted in four urban districts in Tanzania, namely Kinondoni Municipality, Kibaha Town Council, Iringa Municipality and Songea Municipality between January and June 2019. These districts were purposively selected because they had long experience of implementing TIKA scheme in Tanzania.

### Sampling and sampling procedures

Simple random sampling technique was used to select four urban districts which were implementing TIKA. In each district, the study involved the district hospital, two health centres and two dispensaries whereby health centres and dispensaries were selected randomly. The study was conducted in public health facilities only because TIKA was not implemented in non-public health facilities. Purposive sampling technique was utilized to recruit respondents and participants based on their involvement in the implementation of TIKA. Interview guides were developed and tailored to specific categories of respondents. Furthermore, catchment streets surrounding health facilities were included in the study. In these streets, the study involved local government officials, community health workers and members of health facility committees. These officials were purposively selected because of their involvement in the implementation of TIKA scheme. The information collected from these respondents included facilitators and barriers to the implementation of TIKA scheme.

### Data collection techniques

A total of 12 focus group discussions (FGDs) were conducted (3 in each district) with members of the TIKA scheme. Participants in the FGDs were recruited in the involved health facilities on the day of data collection. FGDs comprised 8–12 members; both men and women. The researchers worked with local guides to recruit FGD participants, but the guides were not present when FGDs were conducted. Furthermore, in-depth interviews were conducted with key informants at the national, district and health facility levels. FGDs were conducted at health facility and interviews were conducted in the respondent’s workplace or home. In-depth interviews and FGDs were conducted in Kiswahili language and lasted between 30 and 45 min. The focus of the study was to understand how TIKA scheme was implemented and factors that facilitated and hindered uptake of the scheme. Table 1 is a summary of the respondents involved in this study.

**Table 1:** Categories of Respondents Involved in the Study

S/N	Level of interview	Respondents	Number of interviews
1	National Level	TIKA coordinators	2
2	District Level	District Medical Officers	4
		District Health Secretaries	4
		TIKA Coordinators	4
		Chairs of Council Health Service Boards	4
3	Health facility level	In-charges of health facilities	12
		Hospital secretaries	4
		Chairpersons of health committees	12
4	Community level	Ward Councilors	4
		Ward Executive Officers	4
		Street/Mtaa Executive Officers	8
	**TOTAL**		**62**

Secondary data were collected through document reviews at the district levels. The documents reviewed included district annual plans, minutes of health committees and TIKA registers. Whereas minutes provided information on the process of developing and implementing TIKA scheme in respective districts, district annual plans and TIKA registers provided information on the uptake of the scheme, including enrolment rates.

### Data management and analysis

The data obtained from in-depth interviews, FGDs and reviewed documents were analysed using thematic approach [22]. First, the recorded interviews and FGDs were transcribed verbatim in Kiswahili, but only selected quotes that were used in the study were translated from Kiswahili to English. Second, both researchers read the transcripts in order to understand the depth and breadth of the data set. Third, RK developed a list of initial codes based on the objectives of the study; and the codebook was refined by SM. Then, data were coded manually as per the identified codes. Other themes which emerged during the coding process were added inductively. Fourth, in order to ensure trustworthiness, responses were compared across different types of respondents and across different districts of the study. In addition, findings from the interviews were triangulated with FGDs and summary of documents. Finally, data were summarized and synthesized, keeping the key expressions of respondents as illustrative cases.

## Results

This section presents the findings on the implementation of TIKA scheme in Tanzania. The section is organised into four key themes, which are establishment and management of the scheme, enrolment procedures and premium payment, benefit package and portability of services, and purchasing of healthcare services.

### Establishment and management of TIKA scheme

Within the context of decentralisation in Tanzania, following a pilot study in 1996 and 1998, district councils were given mandate to establish and implement CHF/TIKA schemes. TIKA scheme was essentially backed by the 2001 CHF Act and guided by the Cabinet Directive No. 36 of 2007. However, the implementation of the scheme varied across districts in terms of annual contribution rates and benefit packages. Annual contributions were determined by the respective district, and amounted to TZS 10,000–30,000 (equivalent to $ 5–15 USD per year). An analysis of district health reports and minutes as well as interviews with most district health managers revealed that Council Health Management Teams (CHMTs) had the mandate to initiate a draft of TIKA by-laws in collaboration with the office of the Council Attorney. The draft TIKA by-laws were discussed in the Council Health Service Boards and in Council Social and Economic Committees and Finance Committees; and opinions were gathered from the local community through community meetings. The draft was then endorsed by the Full Council and sent to the respective ministries for approval.

At the national level, the scheme was managed by the then Ministry of Health and Social Welfare (currently Ministry of Health, Community Development, Gender Elderly and Children). The Ministry was responsible for formulating the Policy and coordinating the establishment of TIKA. On the other hand, the Ministry was also responsible for training, supervising, monitoring and implementing the scheme in district councils. The Ministry also provided matching grants to districts through the NHIF management. At the district level, the management of the scheme was under the CHMT on behalf of the office of the District Executive Director (DED). The CHMT is headed by the office of the District Medical Officer (DMO), assisted by the Council Heath Services Board (CHSB). At the level of the facility, TIKA scheme was headed by facility medical officers in-charge. While at the community level, the scheme was managed by the people through Village Councils and health facility committees. The Village Council was responsible for approving by-laws and other decision while the facility heath committees were responsible for supervising, monitoring and approving the decisions made at the facility level.

Interviews with most district health managers revealed that the establishment of TIKA scheme took long time particularly the institution of by-laws. For example, in Kibaha and Kinondoni Municipalities, while the establishment of TIKA started in 2009, the scheme was launched in 2012 and 2015 respectively. According to district health managers, gathering opinions from the community members and ministerial approval of by-laws took the most time, as illustrated by one respondent:

*“The most difficult step in establishing TIKA scheme was gathering opinions from the community members. We spent a lot of time as many community members were not attending meetings. This not only delayed the process but also resulted in many community members lacking knowledge of the scheme*” (District health manager, Kinondoni).

Another respondent added:

* The approval of the by-laws took a long time. This was a legal process and so there were many procedures to follow. After we submitted our by-laws, there were several rounds of comments. To be open, this step took almost a year. We made follow-ups to the Ministry several times, and in most cases, we were told that we were not the only district which had submitted by-laws. This process took a long time* (District health manager, Kibaha).

The findings also revealed that the establishment of TIKA was hampered by inadequate financial resources to facilitate gathering of opinions from stakeholders and sensitization. Indeed, most districts had not allocated budget for TIKA establishment in their annual plans because they thought that it was the responsibility of the NHIF to support districts financially during the establishment of TIKA scheme in their districts, as exemplified by one respondent:

*“TIKA was new in our district and we did not have budget to finance different activities such as community sensitization and collecting opinions from different stakeholders. We received TZS 10,000,000/= (equivalent to 5,000 US dollars) from the NHIF but these funds were not enough. So, we decided to gather public opinions in only few streets* (District health manager, Kinondoni).

Another respondent added:

“*Another important component which was expensive is sensitizing stakeholders, including members of the community. We had limited financial resources to finance all activities. We decided to skip some of the activities*” (District health manager, Iringa).

Apart from limited financial resources, the sensitization process itself was very challenging. For example, it was not easy to explain the benefits of the TIKA scheme to people. This was mainly because some districts such as Kibaha, Iringa and Songea had been implementing the rural based CHF scheme, whose performance proved poor. Thus, community members were not convinced that TIKA would make a difference, as illustrated by some respondents:

“*The main challenge was sensitizing community members; to make people understand that TIKA scheme was important in enabling community to get access to healthcare services. Many community members could not differentiate TIKA from CHF, which had been implemented earlier in their community*” (District health manager, Kibaha).

A similar view was held by another respondent thus;

“*The key challenge was convincing members of the community to join TIKA scheme. The District had since late 1990s been implementing CHF, and the major challenge was shortage of drugs and other services. These largely contributed to low enrolment or renewal of membership. Thus, it has been challenging to convince members of the community to join TIKA as they think it is just the same*” (In-charge of health facility, Songea).

### Enrolment of TIKA members and payment of premium

TIKA was designed to cover urban community groups which were not covered by government or private health insurance schemes in order to reduce the burden of out-of-pocket payments. Initially, enrolment was centrally coordinated by district health management but from 2012, the central government decided to decentralize enrolment and management of funds to dispensaries, health centres and hospitals. Each health facility was directed to open a bank account for managing the funds collected at the health facility. The premium covered an individual; unlike CHF, which covered the family, father, mother and four children. Interviews revealed that, initially, all districts replicated the rural-based community health fund to urban areas. In almost all districts, a member was supposed to pay a premium of 10,000/= Tanzanian shillings (equivalent to 5 US dollars). However, from 2011, the government piloted a new model of CHF called improved (re-designed) community health fund (iCHF). Among the improvements included enrolment by the village enrolment officers, introduction of provider-purchaser split and pooling of funds at the regional level. Other improvements were monthly reimbursement of facilities based on the number of clients receiving healthcare and increased portability of healthcare services across districts and regions. Additionally, annual premium rate increased to TZS 30,000 (equivalent to 15 US Dollars). Consequently, the government issued a circular to all districts to implement iCHF in 2018. This is to say, all districts in Tanzania no longer implemented CHF/TIKA from 2018, but iCHF instead. Despite the new reforms, membership to iCHF remained voluntary in both rural and urban settings. Almost all respondents had the view that the government should consider making health insurance compulsory to all Tanzanians with a view to increasing risk pooling and hence financial resources that can be used to improve the quality of healthcare services. A vast majority of district health managers recommended that the government should fully or partially subsidise individuals who are not able to pay the premium.

### Benefit package and portability of services

Before 2018, benefit package in all the four study districts was limited to one primary healthcare facility where a member had registered; and TIKA cards were not portable across providers. The iCHF pooled funds to the regional level and thus expanded the benefit package and portability of services across districts and regions.

In this study, TIKA registers for 2018 were reviewed, followed by analysis of interviews with most district health managers and in-charges of health facilities. The findings indicated that, in the early years of establishment of TIKA, the enrolment rate was relatively higher. In the subsequent years, however, enrolment started to decrease as many members did not renew their membership mainly due to poor quality of the health services offered in the health facilities, as commented by some respondents:

*When we started implementing TIKA, we sensitized people on the importance of the scheme, and many people joined the scheme. Soon thereafter, we started receiving complaints from members about the quality of healthcare services, especially shortage of medicines. Unfortunately, we had no money to purchase medicines as we had not received matching grants from the central government* (District health manager, Iringa).

*Consequently, members did not get the expected healthcare services. You know, our public health facilities face some challenges, especially shortage of medicines. This situation discouraged people from joining the scheme as well as renewing their membership* (District health manager, Songea).

FGDs with TIKA members in almost all districts confirmed that low enrolment and renewal of membership were mainly caused by poor quality of healthcare services. Members also reported that the package of services offered was limited to primary healthcare services within their respective districts. This meant then that in case members got referral to higher level facilities, they had to cover the costs of the services. In addition, one could not get healthcare services outside the district of residence. However, in Kibaha and Iringa districts, benefit packages were expanded to enable members to receive healthcare services in district and regional hospitals. Nevertheless, many members of the community did not see the importance of joining the scheme or renewing their membership, as commented by some respondents:

*“Many people prefer expanded benefit packages to allow them access to services even beyond their district or region. As a result, some people decide to pay TZS 50,400 (USD 25) for the national health insurance fund (NHIF) for their children instead of TZS 10,000 (USD 5) for TIKA scheme. This is because NHIF has expanded benefit packages compared to TIKA*” (District health manager, Kinondoni).

**Fig. 1:**
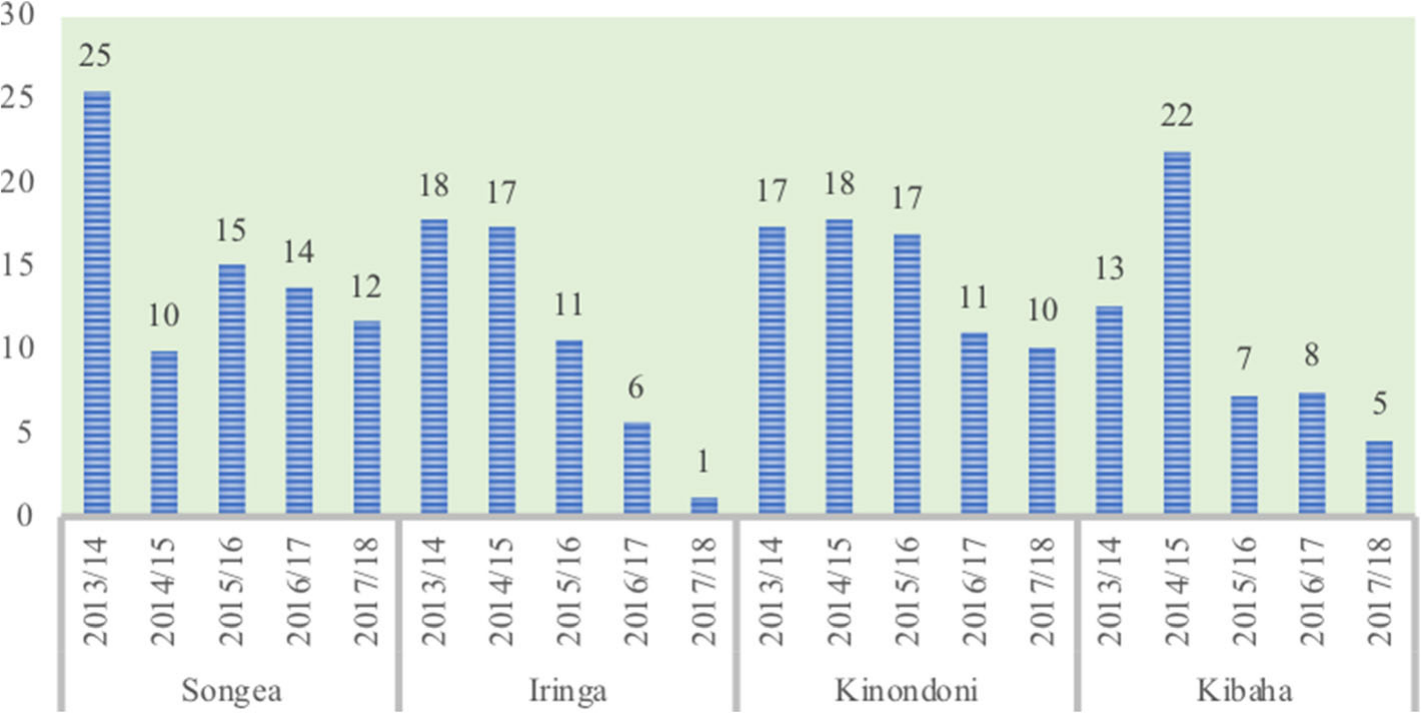
Trends in Enrolment in TIKA. Source: Technical and financial progress implementation reports of Comprehensive Council Health Plans for Kinondoni, Kibaha, Iringa and Songea 2014/15–2017/18

As indicated in Fig. 1 below, enrolment for TIKA and membership had remained persistently low in all study districts.

### Purchasing of healthcare services

Before 2018, TIKA funds reverted back to health facilities through the Comprehensive Council Health Plan (CCHP) activities budgeted from cost-sharing funds. There was no separation between the provider of services and the purchaser as the district authority was both the provider and the purchaser of the services. Payments to health facilities were largely input-based rather than output-based. The review of documents indicated that the government had been implementing a number of health financing reforms aimed at, among others, improving CHF/TIKA. For example, in 2012, the government issued a circular to all districts directing health facilities to open facility bank accounts, which are credited by funds from the insurance reimbursement (NHIF, CHF), user fees and basket funds. Since 2017/2018, the government started the implementation of the Direct Health Facility Financing (DHFF) and Facility Financing and Accounting Reporting System (FFARS). Following the scaling-up of the iCHF since 2018, health facilities will be reimbursed for services provided to the iCHF members (fee per service). In this new arrangement, CHF funds are pooled at the regional level and managed by the Regional Administrative Secretary (RAS).

## Discussion

This study aimed at examining the design and implementation of the community-based health insurance scheme in urban settings in Tanzania. The findings of the study indicate that TIKA scheme faced structural and operational problems which affected the uptake of the scheme. The most important challenge was low uptake of the scheme; which was attributed to the poor quality of healthcare services and limited benefit packages. Structural and operational problems have been frequently reported in earlier assessments of rural-based community health insurance schemes in Tanzania [12–15]. Similar trends have also been reported in other LMICs [9–11].A recent study in Tanzania indicated that exemption policies and healthcare seeking behaviour negatively influence voluntary enrolment in CHF scheme [19]. Earlier studies have also reported that exemption policies potentially discouraged people from joining CHF scheme [12, 19, 23, 24].

With regard to design and management of TIKA scheme, the findings suggested that the voluntary nature of TIKA scheme resulted in many members not renewing their membership after expiry. These findings suggest that although CBHI schemes are one way to organise and mobilise community initiatives, they cannot be expected to provide a major source of funding to finance health systems for UHC. It is understood that the government has drafted ‘the Tanzania Health Financing Strategy 2016–2026: Path towards Universal Health Coverage’ document, whereby some plans have been put in place to reform CHF/TIKA schemes by making it compulsory. However, the Health Financing Strategy has yet to be approved and launched by the government. The government should fast track the implementation of the health financing strategy and ensure that the national health scheme is funded from general government revenue or other forms of taxation that subsidize those unable to pay.

Globally, there is an increasing call to change the voluntary nature of community-based health insurance schemes [18, 25]. A large body of evidence shows that countries which have made significant progress on access and financial protection objectives are those which have successfully implemented mandatory contributions for people who can afford to pay through taxation, and/or compulsory earmarked contributions for health insurance [18, 26]. However, making health insurance mandatory requires adequate legal and regulatory framework to help safeguard the mandatory membership and protect a standard Minimum Benefit Package (MBP) and entitlement of members. Furthermore, the government needs to ensure participation of users in setting contribution levels and modes of payment.

The findings revealed that the decentralized management of the TIKA scheme made it impossible to centrally collect and manage funds, thereby reducing pooling of resources. This situation not only increased fragmentation but also made it difficult for districts to receive matching grants from the central government. The findings thus support the new initiatives of the government which centralize CHF/TIKA scheme at the regional level and transfer the management of the CBHIs to the National Health Insurance Fund. This is because the new initiatives, which were scaled-up across all districts/councils gradually since 2018, have addressed some of the challenges raised in this study. For instance, enrolment is done at the village level by enrolment officers while there is also purchaser-provider split and funds are pooled at the regional level. Moreover, facilities will be reimbursed in their bank accounts each month based on the number of clients receiving care along with portability of benefits across districts and regions. With these new reforms, the councils no longer implement TIKA, but rather the improved community health fund (iCHF). It would be of interest in the future to see how these new initiatives will improve enrolment and access to healthcare services and their contribution to achievement of UHC. In the proposed new health financing strategy, the government plans to merge different health insurance schemes into a single national health insurance. This would help overcome the current fragmented nature of health financing mechanisms in Tanzania thereby promoting universal coverage of healthcare services. Costing studies in Tanzania have shown that in order to make CBHI work, major improvements would be needed, but given the wider health insurance financing context and health seeking behaviours, it is questionable as to whether such improvements are feasible, scalable and valued for money [19, 27].

While within the NHIF there is a directorate to oversee the operations of the CHF/TIKA, there is a need to further centralize the management of the CHF/TIKA from regional to the national level since the current centralization to the regional level does not allow CHF/TIKA cards to be used beyond the region. Furthermore, centralizing the management of CHF/TIKA funds may likely increase resource pooling and the size of revenue collectively available to fund health services. A large body of evidence shows that the larger the degree of risk pooling in a health financing system, the less people will have to bear the financial consequences of their own health risks, and the more likely they are to have access to the care they need [5, 8, 25].

This study largely informs improvements in the ongoing efforts to achieve Universal Health Coverage. The study reiterates that although CBHI schemes are one way to organise and mobilise community initiatives, they cannot be expected to provide a major source of funding to finance health systems for UHC. The major limitation of the study is that it was conducted when the government was in the course of reforming the TIKA scheme. This is the basis of the urge to assess these new initiatives in terms of improved enrolment and access to healthcare services as well as contribution to achievement of UHC.

## Conclusions

This study concludes that both rural and urban-based community health insurance schemes face significant structural and operational challenges which subsequently result into low uptake of the schemes. In order to achieve universal health coverage, the government should consider integrating or merging CBHI schemes into a single national pool with decentralised arms with national support while maintaining local accountability. The national health financing mechanism should be funded from general government revenue that subsidizes those unable to pay. This would increase risk pooling of resources, improve reimbursement process and, in turn, increase the viability of the schemes. There is also a need to strengthen governance at the local level through district and subdistrict level governing bodies to overcome the potential loss of control and participation by lower levels and communities.

## Data Availability

The datasets are not publicly available since participants did not give consent for public sharing of their information. However, summaries of the information are available from the corresponding author upon request. The data collection tools and meeting reports are also available upon request.
